# Outbreak of *Ralstonia* bacteraemia among chronic kidney disease patients in a haemodialysis unit in the Philippines

**DOI:** 10.5365/wpsar.2022.13.4.960

**Published:** 2022-12-27

**Authors:** Denmarc R Aranas, Bernard A Demot, Thea Pamela T Cajulao

**Affiliations:** a Department of Internal Medicine, Baguio General Hospital and Medical Center, Baguio City, Philippines.; b Infection Prevention and Control Committee, Baguio General Hospital and Medical Center, Baguio City, Philippines.

## Abstract

*Ralstonia insidiosa* is an opportunistic pathogen considered an emerging problem among clinically vulnerable populations such as those with chronic kidney disease. This study presents three cases of *Ralstonia* bacteraemia among chronic kidney disease patients in a haemodialysis unit in Baguio City, the Philippines. Case 1 was an elderly male who experienced chills during two concurrent dialysis sessions. Case 2 was a young female who also experienced chills and dizziness during a dialysis session; as this was thought to be related to hypotension, she was admitted. Case 3 was an elderly female with known hypertension and diabetes who had been newly diagnosed with chronic kidney disease; she was brought to the emergency department hypotensive, dyspnoeic and disoriented with deranged laboratory parameters and was admitted to the intensive care unit. All three cases had blood cultures positive for *R. insidiosa* with an attack rate of 1.67%. Drug and device tracing were conducted and environmental samples collected to identify the source of infection. A sample from the faucet of the reprocessing machine in the haemodialysis unit that was positive for *Ralstonia spp*. was the source of the outbreak. Control measures were implemented and the haemodialysis unit was thoroughly cleaned. No further cases were reported, with active surveillance continuing until January 2022. Taken with previously published outbreaks, these findings suggest that medical products and devices can be contaminated with *Ralstonia spp*. and cause illness. Early identification of cases and the source of infection is required to prevent large outbreaks in this vulnerable population.

Nosocomial infection among immunocompromised patients is an emerging problem commonly encountered with multidrug-resistant Gram-negative bacteria. *Ralstonia spp*. are waterborne Gram-negative bacteria, ubiquitous opportunistic environmental pathogens characterized as strong biofilm producers that are resistant to most antimicrobials. Notable strains are *R. pickettii, R. mannitolilytica* and *R. insidiosa*. (*1*)

*R. insidiosa* has recently had increasing clinical relevance, (*2*) especially in hospitals, because it can survive in different ultra- or high-purification water systems used for industrial and laboratory methods. (*3*, *4*) It can contaminate purified or distilled water used for medicinal procedures or products, and can survive in low-nutrient states and be resistant to commonly used antimicrobial agents such as chlorhexidine. (*5*) The emergence of *R. insidiosa* as a causative agent of nosocomial infections was reported among immunocompromised individuals in the Czech Republic, where it led to bacteraemia among eight haemodialysis patients owing to contaminated haemodialysis solutions. (*6*, *7*) A recent report from a Chinese tertiary hospital has noted the emergence of multidrug-resistant  *R. insidiosa* in clinical isolates. (*8*)

In January 2021, the Department of Internal Medicine – Infectious Disease and Infection Prevention and Control Committee (IPCC) in the Philippines declared an outbreak in a haemodialysis unit in Baguio City when three patients were identified with *Ralstonia* bacteraemia. Haemodialysis sessions were suspended until the investigation was completed. The objectives of this study were to describe the three cases of *Ralstonia* bacteraemia and to report the identification process and control measures implemented for this outbreak of *R. insidiosa* in a haemodialysis unit in Baguio City.

## CASE SERIES

In this study, a confirmed case was defined as a patient who underwent haemodialysis and experienced a temperature of more than 38.5 °C or chills during or after the session with a positive blood culture for *R. insidiosa* from December 2020. Clinical histories and laboratory examinations were reviewed for all reported patients. Active surveillance, whereby symptomatic patients from the haemodialysis unit had specimens collected for blood culture and sensitivity testing, was initiated, and continued until January 2022. All specimens underwent sensitivity testing for a range of antibiotics.

Three patients from the haemodialysis unit fit the case definition (**Table 1**). The haemodialysis centre has 30 units catering to 180 dialysis patients; thus, the attack rate was 1.67%.

**Table 1:** Clinical characteristics of three cases of *Ralstonia* bacteraemia detected among chronic kidney disease patients at a single institution in Baguio City, the Philippines, 2020

Characteristics	Case 1	Case 2	Case 3
Onset date	9 December	17 December	20 December
Age/Sex	70/Male	32/Female	62/Female
Comorbidities	Chronic kidney disease, hypertension	Chronic kidney disease, hypertension	Chronic kidney disease, hypertension, diabetes mellitus
Haemodialysis access	Right internal jugular catheter	Right internal jugular catheter	Right internal jugular catheter
Time on haemodialysis	1 year	1 year	2 months
Presenting symptoms	Chills	Chills, hypotension	Disoriented, hypotension, fever
Treatment received for bacteraemia	Co-trimoxazole 800/160 1 tablet once daily for 7 days	Cefepime 500 mg intravenous once daily for 7 days	Co-trimoxazole 800/160 1 tablet once daily for 7 days
Outcome	Discharged	Discharged	Discharged

### Case 1

Case 1 was a 70-year-old male with known stage 5 chronic kidney disease secondary to hypertensive nephrosclerosis. He was on maintenance haemodialysis twice a week at the haemodialysis unit. During a haemodialysis session on 9 December 2020, the patient experienced chills with no associated chest pain or fever. A specimen was collected for blood culture and sensitivity testing before discharge from the haemodialysis unit. Three days later, the patient underwent his next regular haemodialysis with recurrence of chills. After haemodialysis, the patient was sent to the emergency department. He was awake, comfortable, not in distress and had stable vital signs. The patient has an intact right internal jugular catheter. He was sent home and advised to continue maintenance medications and haemodialysis. The blood culture revealed growth of *R. insidiosa*. He was prescribed co-trimoxazole 800/160 one tablet daily for 7 days for the bacteraemia. The patient recovered from the bacteraemia and was discharged.

### Case 2

Case 2 was a 32-year-old female with known stage 5 chronic kidney disease secondary to chronic glomerulonephritis. She was on haemodialysis twice a week. A few hours before admission to the haemodialysis unit on  17 December 2020, she experienced chills, dizziness and body weakness, with hypotension at 80/60 mmHg. A 500 mL fast drip of normal saline solution was given and haemodialysis continued. As the chills and body weakness persisted, the haemodialysis was terminated and the patient was transferred to the emergency department. She was diagnosed with sepsis and admitted to the isolation ward. Her chest tube was removed and the tip sent for culture. Blood samples from two sites were submitted for culture and antibody sensitivity testing. The chest tube tip culture was positive for *Enterococcus faecalis* and the blood culture was positive for bacteraemia with *R. insidiosa*. Cefepime 500 mg intravenous once daily for 7 days was given for the *Ralstonia* bacteraemia. The patient recovered from the bacteraemia and was discharged.

### Case 3

Case 3 was a 69-year-old female with known hypertension and diabetes mellitus. She had a 1-month history of bipedal oedema with decreasing urine output. She reported shortness of breath and progressive bipedal oedema in November 2020. She attended a different hospital where initial tests detected elevated creatinine, after which she was admitted and managed as a newly diagnosed chronic kidney disease patient secondary to hypertensive nephrosclerosis versus diabetic nephropathy. On 20 December 2020, the patient had bradycardia and hypotension at 80/50 mmHg and was given a dopamine drip. She was referred to our institution for haemodialysis the next day.

The patient arrived at the emergency department with symptoms of drowsiness, disorientation and episodes of desaturation at 79% when on room air, and was afebrile. She had anicteric sclera, slightly pale palpebral conjunctiva and positive neck vein engorgement. Her chest findings had crackles in the middle and basal lobes. The patient had bradycardia with irregular rhythm and no murmurs. Extremities had pitting bipedal oedema, grade 3.

The patient was assessed as having acute respiratory failure secondary to encephalopathy, which had resulted from chronic kidney disease, newly diagnosed, and itself the result of hypertensive nephrosclerosis versus diabetic nephropathy, complicated urinary tract infection, pulmonary congestion, metabolic acidosis, multiple electrolyte imbalance and anaemia; uncontrolled stage 2 hypertension; diabetes mellitus type 2, non-obese, non-insulin requiring; and suspected coronavirus disease (COVID-19). The patient was admitted to the intensive care unit for close monitoring and further management, and was then initiated on haemodialysis. A complete blood count revealed increased white blood cells with neutrophilic predominance. Blood culture detected the growth of *R. insidiosa*. Co-trimoxazole 800/160 one tablet daily for 7 days was given for the bacteraemia. The patient recovered from the bacteraemia and was discharged.

### Antibiotic susceptibility testing

Antibiograms of cases 1 and 2 were both resistant to amikacin and gentamicin with sensitivity to most of the other antibiotics tested. Case 2 was also resistant to piperacillin-tazobactam. Case 3 was sensitive or had intermediate results for all antibiotics (**Table 2**).

**Table 2:** Antibiogram of *R. insidiosa* isolates in blood cultures in the three clinical cases of *Ralstonia* bacteraemia detected among chronic kidney disease patients at a single institution in Baguio City, the Philippines, 2020

Antimicrobial	Case 1		Case 2		Case 3	
	MIC (µg/mL)	Interpretation	MIC (µg/mL)	Interpretation	MIC (µg/mL)	Interpretation
Amikacin	^3^64	R	^3^64	R	8	S
Cefepime	2	S	4	S	£1	S
Ceftazidime	16	I	16	I	£1	S
Ciprofloxacin	£0.25	S	£0.25	S	£0.25	S
Gentamicin	^3^16	R	^3^16	R	8	I
Imipenem	2	S	2	S	2	S
Meropenem	4	S	4	S	2	S
Piperacillin/Tazobactam	64	I	^3^128	R	64	I
Trimethoprim/Sulfamethoxazole	£20	S	£20	S	£20	S

I: intermediate; MIC: minimum inhibitory concentration; R: resistant; S: sensitive.

## INVESTIGATION AND CONTROL MEASURES

A review of all drugs and devices used for each case from 15 days before the onset of symptoms until the confirmation of *Ralstonia* bacteraemia was conducted. On 10–15 January 2021, environmental samples were collected from 44 sites throughout the haemodialysis unit, including reprocessing tubing, faucets, suction tubing, suction containers, water sources, venous or arterial site coupling machines and bleach source machines. Samples were also collected from supplies, disinfectants, working areas and devices. All samples were cultured by the hospital’s Department of Pathology for identification to the genus level only. Sensitivity testing was not conducted as per the hospital protocol for environmental samples.

Of the 44 collected samples, 25 were positive for a range of organisms, including: *Ralstonia spp., Aeromonas spp., Pseudomonas aeruginosa, Rhizobium radiobacter, Bacillus spp., Sphingomonas paucimobilis, Pseudomonas putida, Pseudomonas stutzeri, Acinetobacter baumannii, Delftia acidovorans, Serratia plymuthica, Aeromonas hydrophila, Aeromonas punctata, Klebsiella oxytoca*, coagulase-negative *staphylococci, Staphylococcus epidermidis, Staphylococcus haemolyticus* and *Leclercia adecarboxylata* (**Table 3**). The faucet of the reprocessing machine was the only site that was positive for *Ralstonia* species.

**Table 3:** Environmental samples from a haemodialysis unit where *Ralstonia* bacteraemia was detected  among chronic kidney disease patients by site and results at a single institution in Baguio City, the Philippines, 10–15 January 2021

Sites	Growth
1. Faucet, reprocessing machine	*Ralstonia* spp.
2. Reprocessing tubing, station 2	*Aeromonas* spp.
3. Reprocessing tubing, hep c	*Pseudomonas aeruginosa*
4. Reprocessing tubing, hep b	No growth after 48 hours of incubation
5. Water processing machine knobs	*Rhizobium radiobacter*
6. Point of use	No growth after 48 hours of incubation
7. Product tank	No growth after 48 hours of incubation
8. Acid mixer faucet	No growth after 48 hours of incubation
9. Bubbler, station 3	*Bacillus* spp.
10. Oxygen port, station 20	*Sphingomonas paucimobilis*
11. Oxygen port, station 18	No growth after 48 hours of incubation
12. Panasonic refrigerator	*Bacillus* spp.
13. Suction tubing 1	No growth after 48 hours of incubation
14. Suction tubing 2	No growth after 48 hours of incubation
15. Suction container 1	*Pseudomonas putida*
16. Suction container 2	No growth after 48 hours of incubation
17. Suction container 3	No growth after 48 hours of incubation
18. Venous site coupling, machine 30	*Pseudomonas stutzeri*
19. Arterial site coupling, machine 30	*Acinetobacter baumannii*
20. Water source, machine 30	No growth after 48 hours of incubation
21. Citro clean, machine 30	No growth after 48 hours of incubation
22. Bleach source, machine 30	No growth after 48 hours of incubation
23. Chair, machine 30	*Staphylococcus haemolyticus*
24. Venous site coupling, machine 13	No growth after 48 hours of incubation
25. Arterial site coupling, machine 13	No growth after 48 hours of incubation
26. Water source, machine 13	*Delftia acidovorans*
27. Bleach source, machine 13	No growth after 48 hours of incubation
28. Citro clean, machine 13	No growth after 48 hours of incubation
29. Oxygen tank, station 4	No growth after 48 hours of incubation
30. E-cart supply box	No growth after 48 hours of incubation
31. Oxygen port, station 20	No growth after 48 hours of incubation
32. Water source, pantry	*Serratia plymuthica*
33. Pantry sink, faucet	*Aeromonas, hydrophila; Aeromonas punctata; Klebsiella oxytoca*
34. Water dispenser	*Bacillus spp.*
35. Locker handles	*Staphylococcus condimenti*
36. Telephone	*Pseudomonas stutzeri*
37. Keyboard and mouse, station 2	Coagulase-negative staphylococci
38. Keyboard and mouse, station 1	Coagulase-negative staphylococci
39. Medicine table drawer handle	*Staphylococcus epidermidis*
40. Medicine preparation table	*Bacillus spp.*
41. Main door handle	*Staphylococcus haemolyticus*
42. Dialysis stretcher	*Pseudomonas stutzeri*
43. Weight log	***Leclercia adecarboxylata***
44. Working area	***Pseudomonas stutzeri***

Standard and contact infection, prevention and control precautions and disinfection of equipment and the environment were implemented in the haemodialysis unit. The unit was monitored for effectiveness of these preventive measures with follow-up environmental swabs taken to ensure elimination of the source of infection. The wide range of organisms found in the haemodialysis unit indicates the need for maintaining a thorough general cleaning and regular disinfection protocol to prevent opportunistic infections. Upon the results of the environmental testing, thorough disinfection and general cleaning of the haemodialysis unit was conducted.

## Discussion

Three cases of *Ralstonia insidiosa* infection were detected within the haemodialysis unit and were linked to a contaminated faucet in the haemodialysis reprocessing machine. Upon detection of these cases, haemodialysis sessions were suspended and an investigation commenced. Environmental evidence determined the source of infection, after which the faucet of the haemodialysis reprocessing machine was appropriately disinfected and cleaning of the haemodialysis unit was initiated. No further cases have been reported, with active surveillance continuing until January 2022. Several other outbreaks have been reported involving contaminated haemodialysis water as the source of infection. (*9*, *10*)

The low attack rate of 1.67% suggests that the three cases were more vulnerable to infection; however, most patients who require dialysis have similar disease profiles with additional comorbidities and are of older age. The finding that cases had the same access site of the internal jugular haemodialysis catheter does not contribute to increased vulnerability. Right-sided catheters do not relate to increased catheter-related dysfunction and infection. It is therefore possible that they had a greater chance of exposure to the source of infection. (*11*)

Treatment for the three cases in this study was 7 days of cefepime and co-trimoxazole only, given according to the sensitivity of the isolates. In other published outbreaks, most *Ralstonia* infections are treated with ciprofloxacin, amikacin piperacillin-tazobactam, meropenem or a combination of aminoglycosides and cephalosporins with a good response. (*8*, *9*, *12*) There are no current standard recommendations for drugs or duration of treatment of *Ralstonia* bacteraemia. In a report from the Czech Republic, eight cases of central venous catheter infections by *Ralstonia insidiosa* were observed; all isolates from cases had antibiotic sensitivities to β-lactams and fluoroquinolones and were resistant to aminoglycosides. (*12*) Two isolates from this study had similar antibiotic sensitivities to fluoroquinolones, sulfonamide and carbapenems and resistance to aminoglycosides. One case’s isolate had antibiotic sensitivity to almost all drug classes with no resistance.

In conclusion, three patients with chronic kidney disease who required haemodialysis developed bacteraemia with *R. insidiosa*. All three cases had good clinical outcomes after identification of the organism and specific antibiotic treatment. The source of the contamination was identified through environmental testing of possible sites within the haemodialysis unit and was determined to be the faucet of the haemodialysis reprocessing machine. Taken with previously published outbreaks of *Ralstonia spp.*, these findings suggest that medical products and devices can be contaminated with these species and should be suspected when cases are detected. Early identification of these cases and the source of infection is required to prevent large outbreaks and to ensure protection of vulnerable populations such as immunosuppressed patients with end-stage renal disease on haemodialysis.
